# Study and analysis of contralateral acoustic reflex in children with phonological disorder

**DOI:** 10.1590/S1808-86942010000200014

**Published:** 2015-10-19

**Authors:** Tiago Mendonça Attoni, Helena Bolli Mota

**Affiliations:** MSc student in Human Communication Disorders - Federal University of Santa Maria - Speech and Hearing specialist; PhD in Applied Linguistics - Pontifícia Universidade Católica do Rio Grande do Sul, Adjunct Professor of the Speech and Hearing Therapy program for the Universidade Federal de Santa Maria

**Keywords:** hearing, acoustic, child, reflex

## Abstract

Much has been studied on the role of the acoustic reflex in the communication process.

**Aim:**

To examine the responses of the contralateral acoustic reflex in children with normal hearing and phonological disorders. To investigate the relationship of the level of severity of phonological disorder. To measure the chances of it affecting all the frequencies tested.

**Materials and Methods:**

The study was based on the analysis of medical charts from 70 children with phonological disorders, and 24 females and 46 males, aged between 5 and 7 years. Audiological tests were analyzed to exclude children with hearing loss, evaluation of the contralateral acoustic reflex and the level of severity of phonological disorder. Study Design: Prospective.

**Results:**

All children showed change in the contralateral acoustic reflex. There was no significant relationship between the level of severity of phonological disorders and changes in the acoustic reflex for both genders. Female children showed no statistically significant value in the relationship between the frequencies, except at 500 Hz. Male children had more significant relationship in the association between changes in frequencies tested.

**Conclusion:**

It is believed that children with phonological disorders exhibit changes in the contralateral acoustic reflex.

## INTRODUCTION

The acoustic reflex (AR) or stapes muscle reflex is defined as an involuntary contraction of the middle ear muscles in response to a sound stimulus and its recording, which can be done ipsilateral (on the same side of the sound stimulus) or contralateral (on the opposite side of the sound stimulus) to the ear being evaluated^1^. Through acoustic immittance, it is possible to measure the values associated with the AR analyzed in the frequencies of 500, 1000, 2000 and 4000 Hz^2^.

Such analysis has become a major event in audiology, since it allows for the investigation of afferent (sensorial) and efferent (motor) systems which are part of the stapes arc-reflex and that of the tympanic-ossicular system^3^.

AR investigation allows one to check the middle ear all the way to the Superior Olivary Complex.

Many are the functions assigned to AR, such as: improvement in the auditory attention for continuous sounds, separation of the hearing signal from the background noise, perception of intensity alterations above the auditory threshold, damping noises produced by chewing and by mandible movements during speech, participation in vocalization, improvements in speech discrimination under high intensities and frequency selectivity, improvement in localization or sound direction sense through binaural interaction^4^, ^5^, ^6^, ^7^, ^8^, ^9^.

The AR threshold or the intensity level value which guides the normality and integrity pattern of the auditory pathway structures are placed between 70 – 90 dBHL^10^, ^11^; should there be an alteration, one can notice the lack of responses and/or values above 90 dBHL, characterizing AR as altered^12^^,^^13^. In the clinical practice we see patients with normal audiometries, however with AR responses above expected for normality standards and/or lack of responses^14^. These results - where AR alterations happen alone, with the follow up of tonal and vocal audiometry and tympanometry tests within normal patterns have sparkled interest towards research in these areas. Results from audiologic tests within normal ranges can be found in children who undergo speech substitution and omission processes. The speech of these children is characterized by abnormal sound production and inadequate use of the language speech rules according with the disposal or sounds and formation of the syllable structures. These events are called speech deviation or speech disorder^15^. The severity level of the speech disorder is described as the percentage of correct consonants (PCC) produced by the children and classified as severe (PCC<50%), moderate-severe (50%<65%), <100) medium and (65%<85%) medium-moderate >16.

Very little has been unveiled regarding the possibilities of checking AR in children with speech disorders. The Literature is very poor in this issue.

Therefore, considering the information described above, our goals were to check and analyze contralateral AR responses in normal hearing children with speech disorder diagnostic. Moreover, our goal was to investigate whether an AR change in a given frequency is associated with disorders in different frequencies, in the same ear or in the contralateral ear for both genders.

## MATERIALS AND METHODS

This study is registered at the Ethics in Research Committee (CEP) of the institution where it was done, under protocol # 046/02, carried out by means of survey in the Centro de Estudos de Linguagem e Fala (CELF) survey, of a Higher Education Federal Institution. Everyone responsible for the individuals participating in this study was informed about the assessment procedure and they signed an informed consent form, agreeing with the assessment.

As sample inclusion criteria we used the following requisites: have the authorization from the parents or guardians for the child to participate in the study, not have neurological, emotional and/or perception disorders, and/or congenital diseases; be between five and seven years of age; have a satisfactory performance in speech and hearing screening, except those associated with speech and hearing therapy, not have any anatomical or physiological alterations in the speech articulatory organs of expression and understanding language; not have auditory alterations.

The population selected for this study was made up of 70 children, with speech disorder, 20 females and 46 males, with ages between 5 and 7 years.

We used the following equipment:

Fonix FA – 12 clinical audiometer. All the stimuli were deployed in a sound-treated booth by means of a TDH 39 phone (ANSI S3.6/96: ANSI S343/92; ISSO 389/91 calibration).

For the contralateral AR study in the frequencies of 500 to 4000 Hz we used the AZ7 audiometer, TDH 39 phone, with a sound probe sound of 220 Hz to 70 dB (ANSI S3.6/96: ANSI S343/92; ISSO 389/91 calibration).

All the children were submitted to an interview, ear inspection, audiologic evaluation (tonal audiometry, speech reception threshold and speech recognition percentage index), tympanometry, assessment of the speech articulation organs, Child Speech and Hearing Evaluation (AFC)17 and establishing the speech and hearing disorder severity calculated from the PCC16. Moreover, they were submitted to neurological and otorhinolaryngological evaluations.

The 500, 1000, 2000 and 4000 Hz frequencies were tested in the right and left ears in order to assess contralateral AR.

The data was analyzed with the aim of observing which sample incident value of individuals with speech and hearing disorders have contralateral AR alteration. In a second moment, the data was crossed in order to correlate AR alterations with the severity of the speech and hearing disorder. Finally, the data of all the frequencies tested (contralateral) was equally crossed in order to check for a possible relationship that some frequency alteration could have with other frequencies outside the normal range, in the same ear or in a contralateral ear.

The data was statistically analyzed by the Pearson's test for coefficient correlation purposes^18^.

In order to better appreciate the results and discussion, the statistical calculations were also done by gender.

## RESULTS

The study sample showed altered contralateral AR values for the 70 children, in other words, 100% of the results obtained were outside normal ranges for the stapes reflex evaluation. Table 1, Table 2, Table 3 show the acoustic reflex threshold analyses, its distribution and mean value in all the frequencies tested.

**Table 1:** Contralateral acoustic reflex thresholds in the entire sample

Variables	N	mean	SD	VC%	Minimum	Maximum
od500	70	94.92	7.49	7.89	75.0	120
oe500	70	93.78	6.39	6.81	75.0	110
od1000	70	93.21	6.75	7.25	80.0	120
oe1000	70	93.00	6.72	7.23	75.0	110
od2000	70	95.37	8.52	8.94	75.0	120
oe2000	70	94.28	7.33	7.78	80.0	110
od4000	70	94.07	9.06	9.63	80.0	120
oe4000	70	92.14	8.99	9.75	70.0	120

N = subjects

SD = standard deviation

VC% = variation coefficient percentage

**Table 2:** Contralateral acoustic reflex threshold analysis for males.

Variables	N	mean	SD	VC%	Minimum	Maximum
oe500	46	94.78	7.81	8.24	75.0	120
od500	46	93.04	6.62	7.11	75.0	110
od1000	46	93.15	7.25	7.78	80.0	120
oe1000	46	92.93	7.57	8.14	75.0	110
od2000	46	95.32	9.39	9.85	75.0	120
oe2000	46	93.91	7.95	8.46	80.0	110
od4000	46	94.23	10.05	10.66	80.0	120
oe4000	46	92.05	10.46	11.36	70.0	120

N = subjects

DP = standard deviation

VC% = variation coefficient percentage

**Table 3:** Contralateral acoustic reflex threshold analysis for females.

Variable	N	mean	SD	VC%	Minimum	Maximum
od500	24	95.20	6.99	7.34	80.0	110
oe500	24	95.20	5.80	6.09	80.0	105
od1000	24	93.33	5.83	6.25	85.0	110
oe1000	24	93.12	4.84	5.20	85.0	100
od2000	24	95.41	6.74	7.06	85.0	115
oe2000	24	95.00	6.07	6.39	85.0	110
od4000	24	93.75	6.95	7.41	85.0	115
oe4000	24	92.29	5.31	5.75	80.0	105

N = subjects

SD = standard deviation

VC% = variation coefficient percentage

Of the 24 girls, there was no statistically significant value between the level of severity and the speech and hearing disorder in the frequencies tested. Regarding the involvement seen at the given frequencies, there was no significant value which would determine a relationship between the altered frequencies, except for 500 Hz. Having an alteration at 500 Hz, in the left or right ear, together with an alteration in the same frequency in the opposite ear, as depicted on Table 4.

**Table 4:** Results obtained from crossing data between the contralateral acoustic reflex frequencies and the speech disorder severity in females.

Fem	od500	oe500	od1000	oe1000	od2000	oe2000	od4000	oe4000
SD degree	0.037	– 0.084	– 0.132	– 0.097	– 0.105	– 0.097	– 0.133	– 0.012
od500	–	0.695*	0.275	0.268	0.251	0.127	0.117	0.101
oe500		–	0.074	0.285	–0.085	0.030	0.006	0.263
od1000			–	0.115	0.226	0.298	0.274	0.238
oe1000				–	0.224	0.279	0.088	0.131
od2000					–	0.283	0.251	0.280
oe2000						–	0.237	0.204
od4000							–	0.275
oe4000								–

SD degree = speech disorder degree

The (p) significance varied between 0.02 – 0.98 for females.

There was no significant association.

All the frequencies with low significant values.

* Significant at P<.0001

As for the males, the 46 individuals did not show statistically significant values during the testing of the severity level of disorder with alterations in the acoustic reflex. The frequency alterations seen in this gender proved that there is a strong correlation there, on the right or left ears. The disagreement registered in a given frequency is followed by the same alterations in other frequencies. The 4000 Hz frequency in the right ear had a significantly lower value when compared to the other frequencies - when associated with the 1000 and 2000 Hz frequencies. These values are depicted on Table 5.

**Table 5:** Results obtained from crossing data between contralateral acoustic reflex frequencies and the severity of the speech disorder in males.

Male	od500	oe500	od1000	oe1000	od2000	oe2000	od4000	oe4000
SD degree	0.041	0.236	0.638	0.297	–0.058	0.262	–0.119	0.001
od500	–	0.732	0.855	0.583	0.750	0.630	0.584	0.583
oe500		–	0.616	0.792	0.582	0.718	0.536	0.580
od1000			–	0.606	0.726	0.551	0.498	0.570
oe1000				–	0.564	0.755	0.358**	0.665
od2000					–	0.622	0.549	0.609
oe2000						–	0.441*	0.681
od4000							–	0.681
oe4000								–

SD degree = Degree of speech disorder

The (p) significance varied between 0.4817 – 0.9466 for males.

There was no significant association.

All the frequencies were significant at P<.0001

* Significant at P<0.002

** Significant at P<0.01

## DISCUSSION

This paper was possible and very interesting due to the fact that the individuals presented contralateral AR responses which were out of the normal ranges. In fact, abnormal values for these responses and the speech and hearing disorder seem to have a very close relation.

Analyzing children with speech disorders we noticed very different idiosyncrasies and socio-environmental factors among them, leading us to believe that these children must be watched separately, bringing to play numerous approaches and challenges as to etiology and treatment methodology. The inspection and identification of characteristics which are common to this group serve as guiding factor for a better understanding of the factors which lead to the speech disorder and a more efficient treatment approach.

The fact that 100% of the children evaluated showed contralateral AR alterations may serve as a guiding factor for future studies, since the present study represented a common trait among the children with speech disorder.

The results reached in the attempt to associate acoustic reflex alteration with the percentage of correct consonants in the speech of children with speech disorders, did not yield statistical significance indexes, different from one study^19^ which reported an auditory discrimination with the severity of the speech disorder, in which the individuals with medium disorder presented a better performance regarding hearing discrimination, compared to children with a higher speech disorder severity. Another study present in the literature^20^ is the correlation between the speech disorder severity level and the working memory in children, with a positive relation among them.

In comparing the genders, we can notice that there was a prevalence of AR alterations associated with males. We must stress the numerical differences found in the study; nonetheless a very plausible justification for this fact is on the lower rate of people looking for speech therapy for girls, in other words, speech disorders for this gender happen less frequently^21^, ^22^.

As we analyze Table 4, we can notice that there is a lower significant relation regarding the involvement of the frequencies tested, when compared to the values on Table 5. Thus, we must state that among females there are greater chances of having values within what is expected in the normal range for males, which are more susceptible to alterations, observing the relationship existing among the frequencies.

This factor is extremely relevant, since it reaffirms the superiority of females in terms of anatomical and physiological structures associated with speech skills^23^, ^24^.

In girls, we can notice certain flexibility and autonomy as far as AR response analysis goes, in other words, there is no statistically significant value between the frequencies tested in order to state that there is an association between them, their values are somewhat independent. There was a statistically significant value for the 500 Hz frequence. In cases of AR alterations in this frequency, either in the right or left ear, an alteration in the same contralateral frequency is also expected^2^.

Boys seem to have a greater predisposition to alterations in responses related to the efferent pathways^25^, ^26^, ^27^; there is a significant correspondence of the frequencies among each other for altered results found in the contralateral AR. As depicted on Table 5, should there be any alteration in one of the frequencies tested, it happens with mismatches in the remaining frequencies, when it is possible to observe a joint association in the AR frequencies for this gender. Notwithstanding, at 4000 Hz, the right ear presented a significantly lower value when associated with the 1000 and 2000 Hz frequencies of the left ear. We did not find high occurrences of absent values in the frequency of 4000 Hz, which disagrees with another study^14^.

What truly stand out in this study are the answers found in all the children regarding contralateral AR in the 500 Hz frequency. It was possible to notice high significance among the data collected for both genders.

This study leads us to think about the action of the auditory system in sounds outlined by this frequency. It is interesting to analyze this result as a whole, in other words, to relate AR alterations and the speech and hearing processes involved in the development of children with speech disorder. If there is an AR trend to help in frequency selectivity, in the damping control of speech sound low frequencies, favoring the perception of high frequency sounds and separation of the auditory signal from the background sound, for example, it is worth believing the 500 Hz frequency with altered AR value, within a situation such as speech disorder, can decisively impact the speech acquisition process.

In recent studies^14^^,^^28^, we have seen a very close relation between auditory process disorders^29^ and AR alterations in ears without apparent signs of tympanic-ossicular involvement.

In the study about temporal resolution performance in children with speech disorder, the results obtained show that these children are prone to presenting temporal processing and need more time to detect time intervals between auditory stimuli when compared to the children without speech disorders^30^.

In agreement with what has been presented and analyzed, we understand that the AR, as well as its functions and representations in the auditory system, must be investigated more in depth in children with speech disorders.

A future study must propose an investigation of speech disorders, AR recording and the auditory processing, with the aim of truly confirming these relations and discovering important tips of clinical relevance.

## CONCLUSION

All the children assessed presented some contralateral AR alteration and/or its absence; we then suggest a routine exam on AR thresholds in patients with speech disorders.

We did not find statistically significant relation when we tried to associate contralateral AR alterations and speech disorder severity.

The results shown suggest a greater involvement of the contralateral reflex structures in male children. There is a connection between disorders in the frequencies tested for this gender. As far as females go, this is less suggestive of these alterations, except for the 500 Hz frequency in the population studied.

One may think that children with speech disorder who have altered acoustic AR may develop some difficulties regarding auditory skills.

## References

[1] Amaral IEBR, Carvallo RMM. (2008). Limiar e latência do reflexo acústico sob efeito de estimulação contralateral. Rev Soc Bras Fonoaudiol..

[2] Northern JL, Gabbard AS, Kinder DL., KATZ J. (1989).

[3] Carvallo RMM, Mangabeira Albernaz PL. (1997). Reflexos acústicos em lactantes. Acta AWHO..

[4] Simmons FB. (1962). Perceptual theories of middle ear muscle function. J Acoust Soc..

[5] Carmel P, Starr A. (1963). Acoustic and nonacoustic factors modifying middle ear muscle activity in waking cats. J Neurophysiol..

[6] Borg E, Zakrisson JE. (1974). Stapedius reflex and monoaural masking. Acta Otolaryngol..

[7] Liberman MC, Guinan JJ (1998). Feedback control of the auditory periphery: anti-masking effects of middle ear muscles vs. olivochlear efferents. J Commu Disord..

[8] Colletti V, Fiorino F, Verlatog, Carner M. Acoustic reflex selectivity: brain stem auditory evoked response and speech discrimination. In: Katz J. Auditory processing: a transdiciplinary view 1992.p. 39-46.

[9] Wodmald PJ, Rogers C, Gatehouse S. (1995). Speech discrimination in patients with Bells palsy and a paralysed stapedius muscle. Clin Otolaryngol..

[10] Carvallo RMM., Schochat E. (1996).

[11] Metz O. (1952). Threshhold of reflex contraction of muscles of middle ear and recruitment of loudness. Arch Otolaryng. (Chic.).

[12] Castagno LA. (1990). Predição do limiar auditivo através do reflexo estapédico: uma nova fórmula de regressão linear. F Méd..

[13] Carvallo RMM., Pereira LD, Schochat E. (1997).

[14] Marotta RMB, Quintero SM, Marone SAM. (2002). Avaliação do processamento auditivo por meio do teste de reconhecimento de dissílabos em tarefa dicótica SSW em indivíduos com audição normal e ausência do reflexo acústico contralateral. Rev Bras Otorrinolaringol..

[15] Wertzner HF, Amaro L, Galea DES. (2007). Phonological performance measured by speech severity indices compared with correlated factors. São Paulo Med J..

[18] SAS user's guide: statistical, Analysis System Institute, Inc., Cary, NC, 2001.

[19] Santos B (2003). Anais do V Congresso Internacional, XI Congresso Brasileiro de Fonoaudiologia.

[20] Meneguello J, Domenico MLD, Costa MCM, Leonhardt LHFB, Pereira LD. (2001). Ocorrência de reflexo acústico alterado em desordens do processamento auditivo. Rev Bras Otorrinolaringol.

[21] Jerger J. (2000). Diagnosing Auditory Processing Disorders. J Am Acad Audiol..

[22] Muniz LF, Roazzi A, Schochat E, Teixeira CF, Lucena JA. (2007). Avaliação da habilidade de resolução temporal, com o uso do tom puro, em crianças com e sem desvio fonológico. Rev CEFAC..

[23] Linassi LZ, Keske-Soares M, Mota HB. (2005). Habilidades de memória de trabalho e o grau de severidade do desvio fonológico. Pró-Fono..

[24] Freitas GCM. (2001). A consciência fonológica na relação fala-escrita em crianças com desvio fonológico evolutivo. Letras Hoje.

[25] Vieira MG, Mota HB, Keske-Soares M. (2004). Relação entre idade, grau de severidade do desvio fonológico e consciência fonológica. Rev Soc Bras Fonoaudiol..

[26] Harasty J, Double KL, Halliday GM, Kril JJ, McRitchie DA. (1997). Language-associated cortical regions are proportionally larger in the female brain. Arch Neurol..

[27] Schlaepfer TE, Harris GJ, Tien AY, Peng L, Lee S, Pearlson GD. (1995). Structural differences in the cerebral cortex of healthy female and male subjects: a magnetic resonance imaging study. Psychiatry Res..

[28] May BJ, Budelis J, Niparko JK. (2004). Behavioral studies of the olivocochlear efferent system. Arch Otolaryngol Head Neck Surg..

[29] Quaranta N, Scaringi A, Nahum S, Quaranta A. (2005). Effects of efferent acoustic reflex activation on psychoacoustical tuning curves in humans. Acta Oto-Laryngologica..

[30] Anastasio ART, Momensohn-Santos TM. (2005). Identificação de sentenças sintéticas (SSI) e reflexo acústico contralateral. Pró-Fono..

